# Enhancing effect of nicotine on electrical field stimulation elicited contractile responses in isolated rabbit bladder straight muscle; the role of cannabinoid and vanilloid receptors

**DOI:** 10.55730/1300-0144.5527

**Published:** 2022-09-10

**Authors:** Sevil Özger İLHAN, Gökçe Sevim Öztürk FİNCAN, Yağmur OKÇAY, Derya Sebile KOÇ, Celil İlker AŞKIN, Ayşe Kübra KİBAR, İsmail Mert VURAL, Yusuf SARIOĞLU

**Affiliations:** 1 Department of Medical Pharmacology, Faculty of Medicine, Gazi University, Ankara, Turkey; 2 Department of Pharmacology, Gülhane Faculty of Pharmacy, University of Health Sciences, Ankara, Turkey; 3 Department of Medical Pharmacology, Faculty of Medicine, İstinye University, İstanbul, Turkey

**Keywords:** Electrical field stimulation, bladder, nicotine, cannabinoid receptor, vanilloid receptor

## Abstract

**Background/aim:**

Nicotine acts as an agonist of nicotinic acetylcholine receptors (nAChR). These receptors belong to a superfamily of ligand-gated ion channels. We previously demonstrated that nicotine increased electrical field stimulation (EFS)-induced contractile or relaxation responses, possibly by facilitating neurotransmitter release from nerve terminals in various rabbit tissues. Studies have shown that there is an interaction between the endocannabinoid and nicotinic systems. This study aimed to investigate the interaction between nicotine and the endocannabinoid system in the rabbit urine bladder and also investigate the enhancing effect of nicotine on EFS-induced contractile responses in rabbit isolated bladder smooth muscle and its interaction with the endocannabinoid system.

**Materials and methods:**

The New Zealand albino male adult rabbits were used for this study. Following scarification, the urine bladder was rapidly excised, and then uniform strips were prepared. Each strip was mounted under 1 g isometric resting tension in an organ bath containing 20 mL of Krebs–Henseleit solution. After obtaining EFS-induced contractile responses, 10^–4^ M concentrations of nicotine were applied to the preparations, and EFS was stopped after 5 stimulations. Following washing, the same experimental procedure was performed with the same tissue in the presence of AM251 (a cannabinoid CB1R antagonist, 10^–6^ M), AM630 (a cannabinoid CB2R antagonist, 10^–6^ M), and capsazepine (a vanilloid receptor antagonist, 3 **×** 10^–6^ M).

**Results:**

Nicotine enhanced the EFS-induced contraction responses by 17.16% ± 2.81% at a 4-Hz stimulation frequency. Cannabinoid receptor antagonists AM251 and AM630 reduced this increasing effect of nicotine although it was not significant and vanilloid receptor antagonist capsazepine did not significantly alter the nicotines’ effect.

**Conclusion:**

These results show that enhancing effect of nicotine in the smooth muscle of the rabbit bladder, even though it was not significant endocannabinoid system possibly have a role in nicotines’ effect.

## 1. Introduction

Nicotine is an alkaloid that plays an important role in the body and acts as a ligand of nicotinic acetylcholine receptors (nAChRs). In nature, its resource is *Nicotiana tobaccum* plant. Nicotinic acetylcholine receptors are members of pentameric ligand gated ion channels. These receptors’ endogenous ligand is nicotine and binding with nicotine activates the receptors [1]. The pentameric ligand gated ion channel family consists of -HT_3_), γ-aminobutyric acid (GABA) and also nAChRs [2]. nACh receptors consist of two types which are: neuronal receptors and muscular receptors. Neuronal receptors can be found in both central and peripheral nervous systems. These receptors function as a modulator for presynaptic neurotransmitter release. Muscle receptors can be found on neuromuscular junction and they regulate neurotransmitter release at these junctions [3].

The endocannabinoid system consists of cannabinoid receptors (CBRs), synthesizing and inactivating enzymes and a transport protein [4]. Until now, two types of cannabinoid receptors were vastly researched which are CB1R and CB2Rs [5]. CB1 and CB2 receptors were the first receptors that were identified for the marijuana’s active psychotropic ingredient, Δ9-tetrahydrocannabinol (Δ9-THC). This discovery led to the start of the molecular phase of cannabinoid research [6]. Endogenous ligands of CBRs are endocannabinoids. Endocannabinoids are lipid molecules that do not get stored in neuronal vesicles and are released when necessary [7]. There are reviews in the literature that state that there is evidence of endocannabinoids and their effects via cannabinoid receptors on the cardiovascular system, central nervous system and peripheral nervous system [8, 9]. Endocannabinoid receptors are located at both peripheral and central nervous systems at the presynaptic level and these receptors have a modulatory effect on these nerve junctions [10, 11]. CB1Rs are abundantly localized in presynaptic nerve terminals. And this distribution is confirmed with both in situ hybridization [12] and immunohistochemistry [13].

There are proofs which imply for the adjustment of various physiological processes, nicotinic cholinergic and endocannabinoid systems act as regulatory systems [5]. Endocannabinoid and cholinergic systems play an important role in some stages of development of the brain, neuro-adaptive feedback, locomotion control, learning, memory, nociception, reward and some endocrine processes [14–17].

In the literature, there are several studies which demonstrate the relation and interaction between nicotinic, cholinergic and endocannabinoid systems [18, 19]. Receptors of cholinergic and endocannabinoid systems can be found at the same locations and this intertwined distribution of these receptors results in interactions of these two systems. An example of these interactions is cannabinoids’ increasing effects of acetylcholine (Ach) release on nerve terminals [20]. At the hippocampus both synthetic cannabinoids and Δ9-THC (the active ingredient of marijuana) can regulate cholinergic neurotransmission [21, 22]. At presynaptic terminals with the stimulation of nACh receptors and CB1Rs, a modulation for the release of excitatory and inhibitory neurotransmitter occurs; hence, acetylcholine and endocannabinoids alter the synaptic transmission and plasticity in the central nervous system [9, 23].

Several animal studies show that nicotine has an enhancing action on cannabimimetic effects [24]. An animal study demonstrated that when animals are administered with nicotine chronically (1 mg/kg/day for 7 days, s.c.), endocannabinoid levels in their limbic forebrain have increased [25].

There are various familiar effects of delta-9-tetrahydrocannabinol (Δ9-THC) and nicotine, which include antinociception, hypothermia, reward, dependence and loss of quality of locomotion. These can be seen when Δ9-THC and nicotine were given to animals [26–31]. An animal study investigating arachidonylcyclopropylamide (ACPA), which is a synthetic ortholog of N-arachidonylethanolamine (AEA), shows that it has antinociceptive traits. When the ACPA and nicotine were administered together, its analgesic effects are enhanced. In the same study when it is administered with mecamylamine (a nAChR antagonist), ACPA’s analgesic effects are inhibited [32].

These various evidences of interactions between the two systems are mostly can be found in the studies that focused on the central nervous system and the lack of data about these interactions of these two systems at the peripheral tissues motivated us to investigate these interactions at a location other than central nervous system. In our previous studies, we discovered that nicotine enhanced the electrical field stimulation (EFS) elicited contraction or relaxation responses. Nicotine’s enhancing effect on muscle contraction or relaxation responses is per case via its neurotransmitter modulation trait [33–36]. This study aims to investigate the interactions between nicotine and the endocannabinoid system in the rabbit urine bladder.

## 2. Materials and methods

### 2.1. Animals and tissue preparation

A total of six New Zealand albino male adult rabbits (2–3 months old; 2.5–3.0 kg body weight) were used for this study. The animals were fed standard laboratory chow and given tap water ad libitum. This study was approved by the Gazi University Ethics Committee for Animals (G.U.ET-21.019). All animals that used in the study were treated in accordance with the guidelines of the local ethics committee. Rabbits were sacrificed by injecting an overdose of thiopental sodium intravenously (50 mg/kg). The bladder was rapidly excised, opened lengthwise, and emptied. Adherent fat and gross connective tissues were removed, and then uniform strips (20 mm long × 3 mm wide × natural thickness) were prepared [35].

### 2.2. Organ chamber experiments

Each strip was mounted under 1 g isometric resting tension in an organ bath containing 20 mL of Krebs–Henseleit solution (composition in mmol/L : NaCl, 118 ; KCl, 4.7; CaCl_2_·2H_2_O, 1.3; MgCl_2_·6H_2_O, 0.5; Na_2_HPO_4_·2H_2_O, 0.9; NaHCO_3_, 24.9; and glucose monohydrate, 11). The temperature of the solution was 37 °C and after bubbling with 95% O_2_, 5% CO_2_ the pH of the solution was 7.4. Tissues were allowed to equilibrate for at least 1 h before the experimental procedures. EFS was evoked by two parallel platinum electrodes with 4 Hz of stimulation frequencies, trains of impulses of 1 ms duration for 10 s and with a voltage of 60 V in every 2 min by a stimulator (STPT 03, May Research Stimulator; COMMAT Communication Co., Ankara, Turkey) [34]. EFS-induced responses were recorded by isometric force-displacement transducers (FDT10-A, May IOBS 99; COMMAT Communication Co.) connected to an online computer with a data acquisition system (MP30B-CE; BIOPAC Systems Inc., Santa Barbara, CA, USA) using a software (BSL PRO v 3.6.7; BIOPAC Systems Inc.). Before the experimental procedures started, strips were precontracted submaximally with 80 mM potassium chloride (KCl) until the contractile response reached a plateau and after that strips were washed for a duration of 1 h. In order to test the effects of nicotine, preparations were administered with 10^–4^ M concentrations of nicotine [33]. After five contraction responses, EFS were stopped and preparations were washed for a duration of 1 h at every 15th minute. After the wash-out period, EFS was delivered again, and the same experimental procedure was performed with the same tissue in the presence of AM251 (a cannabinoid CB1 receptor antagonist, 10^–6^ M), AM630 (a cannabinoid CB2 receptor antagonist, 10^–6^ M), and capsazepine (a vanilloid receptor antagonist, 3 × 10^–6^ M). Antagonists were added to the organ baths 30 min before the administration of nicotine [37].

### 2.3. Drugs

Nicotine and capsazepine were obtained from Sigma (St Louis, MO, USA), AM251 (1-[2,4-dichlorophenyl]-5-[4-iodophenyl]-4-methyl-N-1-piperidinyl-1H-pyrazole-3-carboxamide) and AM630 ([6-iodo-2-methyl-1-[2-(4-morpholinyl-) ethyl]-1H-indol-3-yl] (4-methoxyphenyl) methanone) were obtained from Tocris (Ellisville, MO, USA).

AM251, AM630, and capsazepine were dissolved in DMSO. Stock solutions of the other drugs were dissolved in distilled water. Solutions were stored at −20 °C until use. The drugs were diluted in distilled water to the required final concentration on the day of use.

### 2.4. Statistical analysis

Nicotine-induced increases were expressed as percentages of the control and the maximum of five EFS-evoked contractile responses. The value of the last contraction before the application of nicotine was taken as the control value. Experimental values were expressed as the mean ± SEM. Groups were compared statistically using Kruskal-Wallis test followed by posthoc analysis with the Dunn’s test. p < 0.05 was considered statistically significant.

## 3. Results

**Figure 1:**
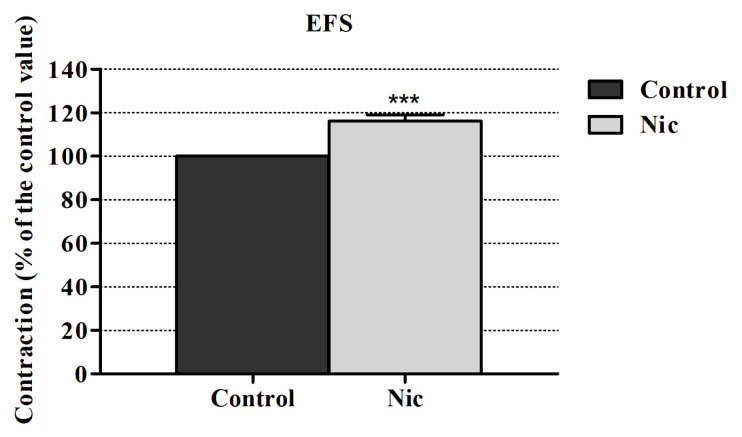
Effect of nicotine (10^–4^ M) on the EFS-induced contractions in the rabbit urine bladder. ***p < 0.0001; significance compared to the control group. Paired t-test was performed (n = 6).

The EFS-induced contraction responses were measured in the rabbit urine bladder. The mean magnitude of EFS elicited contraction amplitudes was 3663.257 ± 343.795 mg. At 10^–4^ M nicotine significantly increased the contraction responses induced by EFS in our study. This enhancing effect of nicotine caused an increase of 17.16% ± 2.81% (p < 0.0001) (Figure 1). These enhancing effects of nicotine were reproducible and after wash-out period, at the second period of EFS, these effects did not differ significantly. During the experiments, no tachyphylaxis was observed.

### 3.1. Effects of cannabinoid receptor antagonists on nicotine’s enhancing effects on EFS-elicited contraction

**Figure 2:**
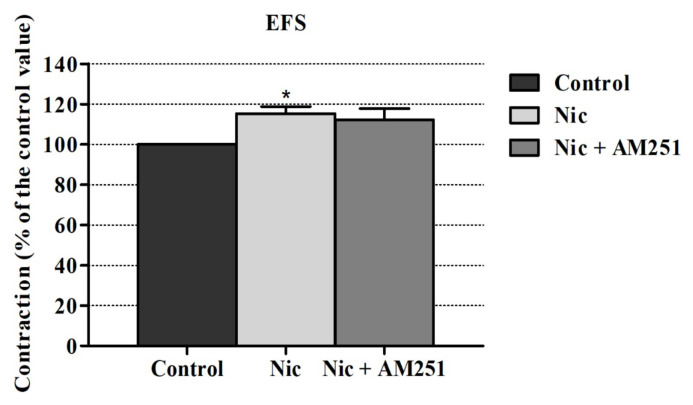
Effect of AM251 on the nicotine-induced EFS contraction enhancement in the rabbit urine bladder. *p < 0.05; significance compared to the control group. Kruskal-Wallis test followed by Dunn’s multiple comparison test was performed using ± SEM values (n = 6).

**Figure 3:**
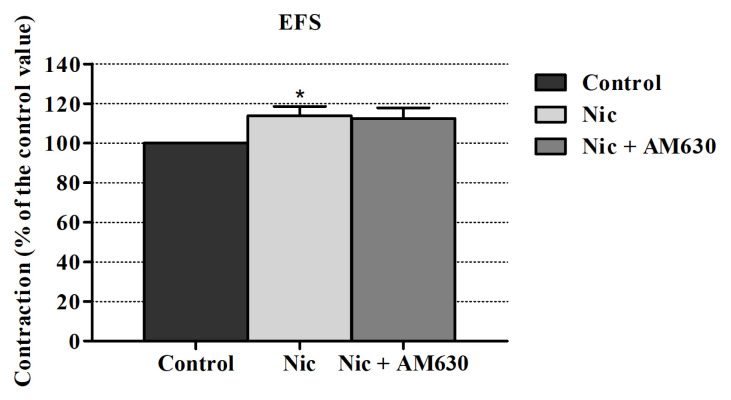
Effect of AM630 on the nicotine-induced EFS contraction enhancement in the rabbit urine bladder. Kruskal-Wallis test followed by Dunn’s multiple comparison test was performed using ± SEM values (n = 6).

AM251 (a CB1R antagonist) inhibited the nicotine’s enhancing effect on EFS induced contraction responses but this inhibition level was not statistically significant. Also, AM630 (a CB2R antagonist) did not alter these responses significantly (Figures 2 and 3).

### 3.2. Effects of vanilloid receptor antagonist on nicotine’s enhancing effects on EFS-elicited contraction

**Figure 4:**
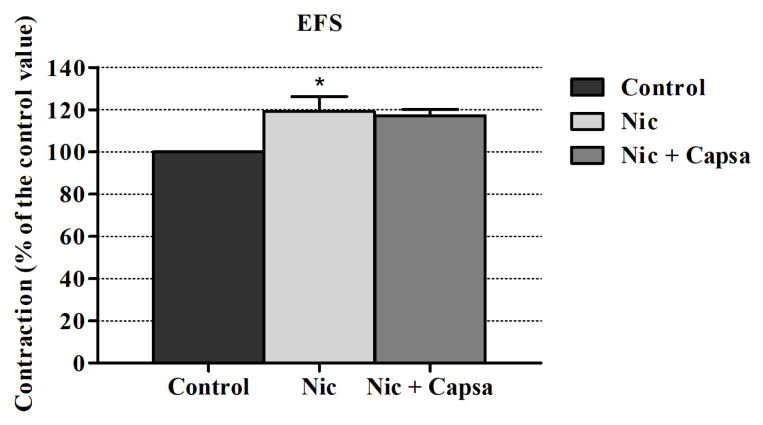
Effect of capsazepine on the nicotine-induced EFS contraction enhancement in the rabbit urine bladder. *p < 0.05; significance compared to the control group. Kruskal-Wallis test followed by Dunn’s multiple comparison test was performed using ± SEM values (n = 6).

Capsazepine (a vanilloid receptor antagonist) did not affect the nicotine’s enhancing effect on EFS induced contraction responses at a significant level (Figure 4).

## 4. Discussion

At 10^–4^ M concentration, nicotine increased the contraction responses caused by EFS in our study. This enhancing effect of nicotine caused an increase of 17.16% ± 2.81%. The mean magnitude of EFS elicited contraction amplitudes was 3663.257 ± 343.795 mg. However, EFS elicited contraction responses and nicotine’s increasing effect on these responses were demonstrated as higher values in our previous study [1]. This result can be caused by the age differences of the animals since in the current study rabbits were 2–3 months old, while in the previous study rabbits were 3–4 months old [38].

Several studies that mostly focused on central nervous system demonstrated that there is an interaction between nicotinic, cholinergic system and endocannabinoid systems. While having modulatory effects on synaptic transmission, endocannabinoids also can act as retrograde messengers at peripheral and central nervous systems. With this trait, it can be said that endocannabinoids can modulate the effects of other neurotransmitters such as acetylcholine. Endocannabinoid receptors which located at the presynaptic nerve terminal regulate the neurotransmitter release when it is stimulated via its ligand which is synthesized from postsynaptic neurons [2, 3]. This information is supported by the knowledge that nACh receptors’ basal activity can be changed with the changes of cell membranes’ lipid composition. This finding can be interpreted as membrane lipids which include AEA, can alter the nACh receptor activity and in some pathological situations with the change of AEA levels at the membranes, can cause substitute levels of activity of nACh receptors [4]

There is evidence that suggests that variations of the α3, α5, α6 and β4 subunits of nAChRs are related to cannabis disorders [5]. Without exact knowledge of how the endocannabinoid and cholinergic systems interact with each other, it can be hypothesized that these interactions can be at cell membrane level or they can interact with each other via second messenger systems. Following this with the stimulation of nAChR or CB1R, conformation of the other receptor could change and alter the affinity of the receptor for its ligand [6].

CB1 receptors at the presynaptic nerve terminals can function as an inhibitor of neurotransmitter transportation to synaptic junction [7] and CB1Rs can activate various subtypes of K^+^ channels and can inhibit voltage-sensitive N-type and P/Q-type Ca^2+^ channels. These results show that CB1Rs have connections to ion channels and these receptors can alter ion channels’ activities via Golf protein [8]. Since nAChRs are ligand gated ion channels it is possible that endocannabinoids can alter the activity of nAChRs via this mechanism. Also when the interactions between nicotinic receptors and endocannabinoids are examined, it can be said that there can be a possible modulatory role of postsynaptic released endocannabinoids on the α7 nAChRs [9]. At the hippocampal interneurons α7 nAChRs and CB1 receptors are localized together. This distribution is confirmed with a double in situ hybridization study [10]. A study using Xenopus oocytes which contain α7 nACh receptors showed that endocannabinoids can act directly on these receptors [11]. Acquas et al. stated that in the central nervous system at the hippocampal level, cannabinoid agonists caused an increase in cholinergic neurotransmission activity and there are other studies in the literature that support these findings [8, 12]. A study by Guo et al. demonstrated that endocannabinoid CB1R can be found on mice hypothalamus and nicotine has an enhancing effect on these CB1Rs’ expression [13]. Even though the exact level of effects of endocannabinoids on nicotinic acetylcholine receptors are unknown, with the current findings on the interactions between these two systems, it can be said that they have an intertwined relationship, especially at central nervous system and possibly, they can mediate and regulate their actions of each other.

Studies suggest that nicotine’s rewarding effects are related to CB1Rs. Some of the evidence which regards nicotine’s behavioral effects are: CB1R knockout mice do not develop the increased locomotor activity levels which can normally be initiated by nicotine [14] and also these mice do not develop the nicotine-initiated conditioned place preference [14, 15]. When nicotine is administered, it can cause behavioral responses with its effects on several locations in central nervous system. It is demonstrated that agonists of cannabinoid receptors can regulate the acetylcholine levels via activating or inhibiting its release and turnover. Therefore, cannabinoids can alter nicotine’s effects on behavioral responses [16–18] also an animal study that uses mice demonstrated that CB1Rs and CB2Rs are associated with nicotine’s behavioral effects [19]. A study revealed that activation of CB1R increases the self-administration of nicotine [20]. When Δ9-THC is administered, it can reduce the severity of nicotine withdrawal signs [21]. With these findings, it can be hypothesized that CB1Rs are related to the enhancement of nicotine seeking motivation and also relapses of this behavior [22]. These interactions can be thought of as it can be occur in both ways since, when cannabis is administered to the body it has a high psychological effect and it also affects heart rate and a clinical trial demonstrated that transdermal nicotine administration increased these effects of cannabis [23]. In line with these results concerning nicotine and endocannabinoid interactions on nicotine dependence, it can be said that the endocannabinoid system has a role in the development of nicotine dependence. Endocannabinoids’ relation with the motivation of nicotine seeking behavior and reward systems could be related to disorders possibly affiliated with reward and behavioral mechanisms such as drug abuse, obesity could be dependent on the endocannabinoid system. Therefore, endocannabinoid system can be a potential treatment target for such conditions [24].

In the literature, it can be seen that several studies revealed that the endocannabinoid system has an effect on the bladder hence the urogenital system [25, 26]. Also, many studies addressed the expression and role of nAChR’s in bladder tissue [27, 28]. Peripheral tissues are also known to express CB1 and CB2 receptors along with nAChR’s. Therefore, an interaction between these receptors on the peripheral level may present another research area. In our study, although not significant, nicotine-induced EFS contraction response enhancement was inhibited by CB1 receptor antagonist AM251 and CB2 receptor antagonist AM630. Vanilloid receptor antagonist capsazepine did not alter these responses significantly. Some limitations to our study were that our sample number (n number) in the current study was 6, this can be expanded in further studies. Also, concentrations of AM251 and AM630 could be increased in additional studies. New studies are needed to demonstrate the exact mechanism of the interactions between these two systems.

## 5. Conclusion

In the present study, nicotine increased the amplitudes of EFS-induced contraction responses in rabbit urine bladder. In our study CB1 receptor antagonist AM251 and CB2 receptor antagonist AM630 inhibited the nicotine’s enhancing effect on EFS induced contraction responses; however, the level of inhibition was not statistically significant. Vanilloid receptor antagonist capsazepine did not alter these responses significantly. It can be concluded that cannabinoids may possibly have a role in nicotine’s actions on the body and, nicotinic and endocannabinoid systems have an interaction between themselves on various physiological processes. However, at what level these interactions occur, and the role of endocannabinoids’ effects on nicotine pathways or vice versa should be investigated in further studies.

## References

[1] Ozturk Fincan GS Vural IM Ercan ZS Sarioglu Y Enhancement effects of nicotine on neurogenic relaxation responses in the corpus cavernosum in rabbits: the role of nicotinic acetylcholine receptor subtypes European Journal Pharmacology 2010 627 1–3 281 284 10.1016/j.ejphar.2009.10.042 19878666

[2] Degroot A Nomikos GG In vivo neurochemical effects induced by changes in endocannabinoid neurotransmission Current opinion in pharmacology 2007 7 1 62 68 10.1016/j.coph.2006.11.001 17174603

[3] Schlicker E Kathmann M Modulation of transmitter release via presynaptic cannabinoid receptors Trends in pharmacological sciences 2001 22 11 565 572 10.1016/s0165-6147(00)01805-8 11698100

[4] Baenziger JE Morris ML Darsaut TE Ryan SE Effect of membrane lipid composition on the conformational equilibria of the nicotinic acetylcholine receptor Journal of Biological Chemistry 2000 275 2 777 784 10.1074/jbc.275.2.777 10625607

[5] Donvito G Muldoon PP Jackson KJ Ahmad U Zaveri NT Neuronal nicotinic acetylcholine receptors mediate Δ(9) -THC dependence: Mouse and human studies Addiction Biology 2020 25 1 e12691 10.1111/adb.12691 30378732 PMC6509006

[6] Oz M Al Kury L Keun-Hang SY Mahgoub M Galadari S Cellular approaches to the interaction between cannabinoid receptor ligands and nicotinic acetylcholine receptors European journal of pharmacology 2014 731 100 105 10.1016/j.ejphar.2014.03.010 24642359

[7] McPartland JM Blanchon DJ Musty RE Clinical Study: Cannabimimetic effects modulated by cholinergic compounds Addiction Biology 2008 13 3 – 4 411 415 10.1111/j.1369-1600.2008.00126.x 18782385

[8] Acquas E Pisanu A Marrocu P Goldberg SR Di Chiara G Delta9-tetrahydrocannabinol enhances cortical and hippocampal acetylcholine release in vivo: a microdialysis study European Journal Pharmacology 2001 419 2–3 155 161 10.1016/s0014-2999(01)00967-0 11426837

[9] Oz M Zhang L Ravindran A Morales M Lupica CR Differential effects of endogenous and synthetic cannabinoids on α7-nicotinic acetylcholine receptor-mediated responses in Xenopus oocytes Journal of Pharmacology and Experimental Therapeutics 2004 310 3 1152 1160 10.1124/jpet.104.067751 15102930

[10] Morales M Hein K Vogel Z Hippocampal interneurons co-express transcripts encoding the alpha7 nicotinic receptor subunit and the cannabinoid receptor 1 Neuroscience 2008 152 1 70 81 10.1016/j.neuroscience.2007.12.019 18222041 PMC2574619

[11] Oz M Ravindran A Diaz-Ruiz O Zhang L Morales M The endogenous cannabinoid anandamide inhibits α7 nicotinic acetylcholine receptor-mediated responses in Xenopus oocytes Journal of Pharmacology and Experimental Therapeutics 2003 306 3 1003 1010 10.1124/jpet.103.049981 12766252

[12] Pisanu A Acquas E Fenu S Di Chiara G Modulation of Delta(9)-THC-induced increase of cortical and hippocampal acetylcholine release by micro opioid and D(1) dopamine receptors Neuropharmacology 2006 50 6 661 670 10.1016/j.neuropharm.2005.11.023 16427098

[13] Guo T Tanaka T Matsumoto M Kaneko K Unzai T A combination of dietary fat intake and nicotine exposure enhances CB1 endocannabinoid receptor expression in hypothalamic nuclei in male mice Neuroscience letters 2020 714 134550 10.1016/j.neulet.2019.134550 31634502

[14] Livingstone PD Wonnacott S Nicotinic acetylcholine receptors and the ascending dopamine pathways Biochemical pharmacology 2009 78 7 744 755 10.1016/j.bcp.2009.06.004 19523928

[15] Merritt LL Martin BR Walters C Lichtman AH Damaj MI The endogenous cannabinoid system modulates nicotine reward and dependence Journal of Pharmacology and Experimental Therapeutics 2008 326 2 483 492 10.1124/jpet.108.138321 18451315 PMC2746999

[16] Acquas E Pisanu A Marrocu P Di Chiara G Cannabinoid CB(1) receptor agonists increase rat cortical and hippocampal acetylcholine release in vivo European Journal Pharmacology 2000 401 2 179 185 10.1016/s0014-2999(00)00403-9 10924924

[17] Gessa GL Casu MA Carta G Mascia MS Cannabinoids decrease acetylcholine release in the medial-prefrontal cortex and hippocampus, reversal by SR 141716A European Journal Pharmacology 1998 355 2–3 119 124 10.1016/s0014-2999(98)00486-5 9760025

[18] Tripathi HL Vocci FJ Brase DA Dewey WL Effects of cannabinoids on levels of acetylcholine and choline and on turnover rate of acetylcholine in various regions of the mouse brain Alcohol and Drug Research 1987 7 5–6 525 532 3620017

[19] Pekala K Michalak A Kruk-Slomka M Budzynska B Biala G Impacts of cannabinoid receptor ligands on nicotine- and chronic mild stress-induced cognitive and depression-like effects in mice Behavioural Brain Research 2018 347 167 174 10.1016/j.bbr.2018.03.019 29551733

[20] Gamaleddin I Wertheim C Zhu AZ Coen KM Vemuri K Cannabinoid receptor stimulation increases motivation for nicotine and nicotine seeking Addiction Biology 2012 17 1 47 61 10.1111/j.1369-1600.2011.00314.x 21521420

[21] Aso Pérez E Maldonado R Murtra P Balerio GN Berrendero Díaz F Delta9-tetrahydrocannabinol decreases somatic and motivational manifestations of nicotine withdrawal in mice European Journal of Neuroscience 2004 20 2737 2748 10.1111/j.1460-9568.2004.03714.x 15548217

[22] Gamaleddin IH Trigo JM Gueye AB Zvonok A Makriyannis A Role of the endogenous cannabinoid system in nicotine addiction: novel insights Frontiers in Psychiatry 2015 6 41 10.3389/fpsyt.2015.00041 25859226 PMC4373509

[23] Penetar DM Kouri EM Gross MM McCarthy EM Rhee CK Transdermal nicotine alters some of marihuana’s effects in male and female volunteers Drug and alcohol dependence 2005 79 2 211 223 10.1016/j.drugalcdep.2005.01.008 16002030

[24] Scherma M Fadda P Foll BL Forget B Fratta W The endocannabinoid system: a new molecular target for the treatment of tobacco addiction CNS & Neurological Disorders-Drug Targets (Formerly Current Drug Targets-CNS & Neurological Disorders) 2008 7 5 468 481 10.2174/187152708786927859 PMC3821699 19128204

[25] Hedlund P Cannabinoids and the endocannabinoid system in lower urinary tract function and dysfunction Neurourology and urodynamics 2014 33 1 46 53 10.1002/nau.22442 24285567

[26] Christie S Brookes S Zagorodnyuk V Endocannabinoids in Bladder Sensory Mechanisms in Health and Diseases Frontiers in Pharmacology 2021 12 708989 10.3389/fphar.2021.708989 34290614 PMC8287826

[27] Beckel JM Birder LA Differential expression and function of nicotinic acetylcholine receptors in the urinary bladder epithelium of the rat The Journal of Physiology 2012 590 6 1465 1480 10.1113/jphysiol.2011.226860 22250215 PMC3382334

[28] Nandigama R Ibañez-Tallon I Lips K Schwantes U Kummer W Expression of nicotinic acetylcholine receptor subunit mRNA in mouse bladder afferent neurons Neuroscience 2013 229 27 35 10.1016/j.neuroscience.2012.10.059 23131712

